# Polysaccharide, the Active Component of *Dendrobium officinale*, Ameliorates Metabolic Hypertension in Rats *via* Regulating Intestinal Flora-SCFAs-Vascular Axis

**DOI:** 10.3389/fphar.2022.935714

**Published:** 2022-07-11

**Authors:** Bo Li, Hui-Ying Wang, Jia-Hui Huang, Wan-Feng Xu, Xiao-Jie Feng, Ze-Ping Xiong, Ying-Jie Dong, Lin-Zi Li, Xinglishang He, Han-Song Wu, Ke Zhang, Jie Su, Qiao-Xian Yu, Ning-Hua Jiang, Gui-Yuan Lv, Su-Hong Chen

**Affiliations:** ^1^ Zhejiang University of Technology, Hangzhou, China; ^2^ Zhejiang Chinese Medical University, Hangzhou, China; ^3^ Zhejiang Senyu Co., Ltd, Yiwu, China; ^4^ The Second Affiliated Hospital of Jiaxing University, Jaxing, China

**Keywords:** *Dendrobium officinale* polysaccharide, metabolic hypertension, short chain fatty acids, G protein-coupled receptor, strengthened enterogastric function

## Abstract

Metabolic hypertension (MH) is the most common type of hypertension worldwide because of unhealthy lifestyles, such as excessive alcohol intake and high-sugar/high-fat diets (ACHSFDs), adopted by humans. Poor diets lead to a decrease in the synthesis of short-chain fatty acids (SCFAs), which are produced by intestinal flora and transferred by G protein-coupled receptors (GPCRs), resulting in impaired gastrointestinal function, disrupted metabolic processes, increased blood pressure (BP), and ultimately, MH. It is not clear whether *Dendrobium officinale* polysaccharide (DOPS) can mediate its effects by triggering the SCFAs-GPCR43/41 pathway. In this study, DOPS, with a content of 54.45 ± 4.23% and composition of mannose, glucose, and galacturonic acid at mass percentages of 61.28, 31.87, and 2.53%, was isolated from *Dendrobium officinale*. It was observed that DOPS, given to rats by intragastric administration after dissolution, could lower the BP and improve the abnormal lipid metabolic processes in ACHSFD-induced MH rats. Moreover, DOPS was found to increase the production, transportation, and utilization of SCFAs, while improving the intestinal flora and strengthening the intestinal barrier, as well as increasing the intestinal levels of SCFAs and the expression of GPCR43/41. Furthermore, DOPS improved vascular endothelial function by increasing the expression of GPCR41 and endothelial nitric oxide synthase in the aorta and the nitric oxide level in the serum. However, these effects were all reversed by antibiotic use. These findings indicate that DOPS is the active component of *Dendrobium officinale*, and it can reverse MH in rats by activating the intestinal SCFAs-GPCR43/41 pathway.

## Highlights

- *Dendrobium officinale* polysaccharide (DOPS) is the active component of *Dendrobium officinale* (DO), which has beneficial effects on rats with metabolic hypertension (MH).
- DOPS can increase the production of intestinal SCFAs, along with improving the intestinal flora and strengthening the intestinal barrier in the MH rats.
- The effect of DOPS on antihypertension may be by regulating the intestinal SCFA-GPCR43/41 pathway of the intestinal flora-SCFAs-vascular axis.

## Introduction

With a high morbidity rate and fast induction rate, hypertension is one of the most common cardiovascular diseases to seriously endanger the health of humans, and it requires life-long medication use. Presently, it is a serious public health problem worldwide (Zhu et al., 2013), which includes countries such as China. According to the most recent nationwide hypertension survey of adults in China, the prevalence of hypertension was 27.9% from 2012 to 2015 (Wang et al., 2018). In China, the estimated total number of individuals with hypertension is 244.5 million. Compared with an earlier survey conducted in 2002, the incidence of hypertension increased by 48.4% (Li et al., 2005; Wang, 2018). Furthermore, more than 60% of the risk factors for hypertension are associated with metabolic disturbances, which inadvertently increase hypertensive risk and cause high blood pressure (BP) (Zhu et al., 2016). Due to the clinical significance of metabolic abnormalities, which must be reversed to bring down the high mortality rate, in the pathogenesis of hypertension, the concept of metabolic hypertension (MH) was proposed (Zhu et al., 2013).

With changes in modern lifestyles, unhealthy diets that include alcohol (Puddey et al., 2019), unhealthy fats (Taylor et al., 2018), and sugar (Roberts et al., 2000) are widespread. Excessive alcohol intake and high-sugar/high-fat diets (ACHSFDs) have been reported to be the major cause of MH, whose treatment and prevention are critical for the treatment of hypertension. In an earlier study, we established an ACHSFD rat model to simulate MH in humans and reported an increased BP, which coincided with abnormal serum lipid levels and liver function (Chen et al., 2015; Li et al., 2021).

The pathological basis of MH is unknown. Food, including alcohol, fats, and sugar, passes through the human body through the gastrointestinal (GI) tract, suggesting that the GI tract can be the initial organ of MH onset (Zhu et al., 2016; Lei et al., 2019). In addition, GI intervention can improve or reverse vascular dysfunction and MH (Zhu et al., 2016). Intestinal flora dysbiosis and intestinal tight junction protein barrier (ZO-1, occludin, and claudin) damage, the important components of the GI function, could contribute to the development of hypertension (Li et al., 2017; Li et al., 2021). A significant decrease in microbial richness, diversity, and evenness was observed in spontaneously hypertensive rats and humans, in addition to an increased Firmicutes/Bacteroidota (F/B) ratio. These changes were accompanied by a decreased abundance of acetate- and butyrate-producing bacteria (Yang et al., 2015); the MH rats also showed intestinal flora disturbance and intestinal tight junction protein barrier damage (Li et al., 2021).

Short-chain fatty acids (SCFAs), with the beneficial effects on intestinal flora and intestinal tight junction protein barrier, are intestinal microbial metabolites that bind to GPCR43/41 receptors for transport throughout the body, thereby also affecting the physiology of hosts. ACHSFD can significantly decrease SCFAs’ concentrations and receptor expression levels as well as disrupt the intestinal flora and intestinal tight junction protein barrier (Agus et al., 2016; Lei et al., 2019). Furthermore, GPCR41, which can be activated by SCFAs, is also expressed by vascular cells, where it relaxes the aorta and lowers the BP. A previous study has reported that oral administration of SCFAs affected the BP in GPCR41-deficient mice (Miyamoto et al., 2016), and GPCR41 knockout mice developed vasogenic hypertension (Natarajan et al., 2016), which was related to the endothelial nitric oxide synthase (eNOS)-nitric oxide (NO) pathway of vascular endothelial function (Li et al., 2021). Therefore, SCFAs elicit vasodilator and anti-hypertensive effects by activating the SCFA-GPCR41 pathway of the intestinal flora-SCFAs-vascular axis.

*Dendrobium officinale* is a traditional Chinese medicine (TCM) used as both a medicine and a food. Shen Nong’s Herbal Classic, an ancient TCM book, has recorded that *Dendrobium officinale* has obvious benefits in the GI function after long-term use, which has been corroborated by modern pharmacological studies (Lei et al., 2019; Li et al., 2021; Yang et al., 2021). For instance, it has been reported that *Dendrobium officinale* can reduce BP in rats with Alcohol-induced MH (Lv et al., 2013). We also reported that *Dendrobium officinale* ultrafine powder (DOFP) could improve the intestinal flora and increase the SCFA level in feces and serum, thereby activating the SCFA-GPCR43/41 pathway, improving endothelial function, and lowering BP in rats with MH, which provides new insights on the mechanism of *Dendrobium officinale* in the treatment of MH (Li et al., 2021). Polysaccharides, as the main medicinal ingredient of *Dendrobium officinale*, have gained increased research interest recently (Lei et al., 2019; Zhang et al., 2020; Lei et al., 2021). However, it is not clear whether *Dendrobium officinale* polysaccharide (DOPS) can also improve the intestinal flora and SCFAs and then activate the intestinal SCFA-GPCR43/41 pathway, which can improve vascular function and lower the BP.

In this study, DOPS was isolated from DOFP, and based on the characterization of DOPS against ACHSFD-induced MH, we further studied and verified the mechanism of action of DOPS against MH by activating the intestinal SCFA-GPCR43/41 pathway, which associates with the roles of intestinal flora and SCFAs. Meanwhile, we examined whether the effects could be reversed by the antibiotics.

## Materials and Methods

### Chemicals and Reagents

The standard reagents of total cholesterol (TC), triglyceride (TG), alanine transaminase (ALT), aspartate transaminase (AST), high-density lipoprotein cholesterol (HDL-c), and low-density lipoprotein cholesterol (LDL-c) were purchased from NingBo MedicalSystem Biotechnology Co., Ltd. (Ningbo, Zhejiang, China). Hematoxylin and Eosin (H&E), Oil Red O solution, DAB chromogenic kit, and Nitric Oxide (NO) Assay Kit were obtained from Nanjing Jiancheng Bioengineering Institute (Nanjing, Jiangsu, China). The antibodies of occludin, claudin, ZO-1, GAPDH, and β-actin were purchased from Proteintech Co., Ltd. (Chicago, United States). The antibodies of eNOS, GPCR41, and GPCR43 were purchased from Hangzhou Hua’an Biotechnology Co., Ltd. (Hangzhou, Zhejiang, China). Reagents for SDS-PAGE were purchased from Beyotime Biotechnology Co., Ltd. (Shanghai, China). The standard reagents for SCFAs were purchased from Dr. Ehrenstorfer Co., Ltd. (Germany). The metronidazole tablets were purchased from Huazhong Pharmaceutical Co., Ltd. (Hubei, China). The azithromycin tablets were purchased from Sunflower Pharmaceutical Group (Hengshui) Derfel Co., Ltd. (Hubei, China). The levofloxacin tablets were purchased from Daiichi Sankyo Pharmaceutical (Beijing) Co., Ltd. (Beijing, China). *Dendrobii officinalis* (DO) stem was obtained from Zhejiang Senyu Co., Ltd. (Zhejiang, China). Plant materials were identified by Professor Ping Wang, School of Pharmacy, Zhejiang University of Technology.

### The Total Polysaccharide Content Analysis of *Dendrobium officinale* Polysaccharide

**FIGURE 1:**
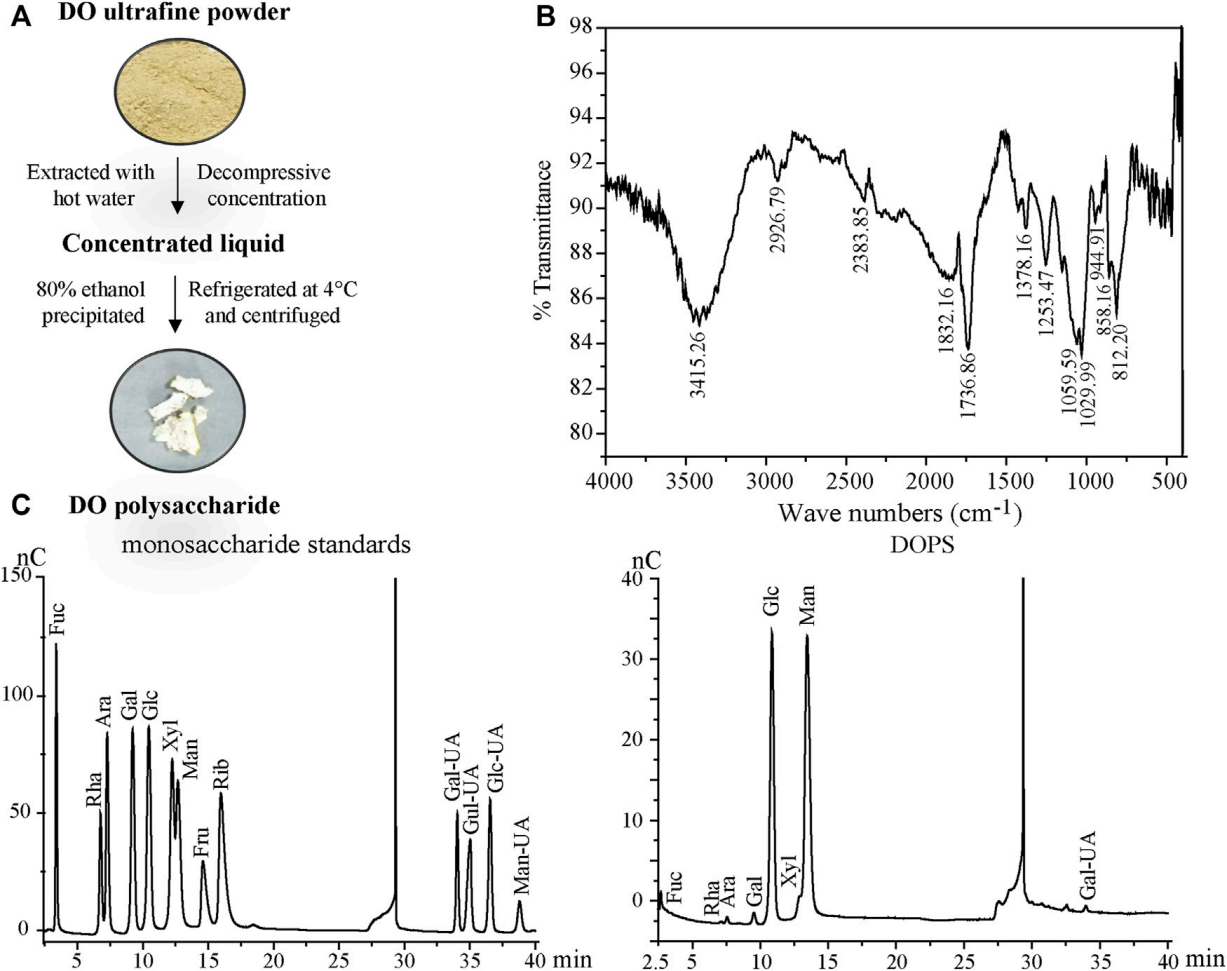
Preparation and properties of DOPS. **(A)** Preparation flow diagram of DOPS. **(B)** Analysis of DOPS by Fourier transform infrared spectroscopy (FT-IR). **(C)** High-performance liquid chromatography (HPLC) analyses of monosaccharide standards and DOPS. DO: *Dendrobium officinale*; Fuc: Fucose; Rha: Rhamnose; Ara: Arabinose; Gal: Galactose; Glc: Glucose; Xyl: Xylose; Man: Mannose; Fru: Fructose; Rib: Ribose; Gal-UA: Galacturonic acid; Gul-UA: Glucuronic acid; Glc-UA: Mannuronic acid; Man-UA: Guluronic acid.

The DOPS used in this study had a similar chemical preparation according to our previous literature (Yan et al., 2015) (Figure 1A). The total polysaccharide content of DOPS was measured by the phenol-sulfuric acid method, having a maximum absorbance of 488 nm. The method was described in our previous research (Lei et al., 2019).

### Infrared Spectroscopy Analysis

DOPS was finely weighed and ground evenly with KBr powder at a ratio of 1:20. Then, the tablets were pressed and analyzed by a Fourier transform infrared (FT-IR) spectrophotometer (Nexus, Thermo, United States) with a scanning range of 4000 cm^−1^—400 cm^−1^.

### Monosaccharide Composition Identification With High-Performance Liquid Chromatography

DOPS was hydrolyzed with trifluoroacetic acid (TFA) at 120°C for 2 h. Then, blow-dry with nitrogen and add methanol for cleaning, repeat 2–3 times. Then, the hydrogenated product and thirteen monosaccharides (Fuc, Rha, Ara, Gal, Glc, Xyl, Man, Fru, Rib, Gal-UA, Gul-UA, Glc-UA, and Man-UA) were dissolved in sterile water and transferred to a chromatographic bottle for testing. Finally, the aqueous phase was filtered and analyzed by the Thermo ICS5000 ion chromatography system (Thermo Fisher Scientific, United States) using a Dionex™ CarboPac™ PA20 column and detected by an electrochemical detector. The mobile phase displayed of high-performance liquid chromatography (HPLC) in Table 1.

**TABLE 1:** Mobile phase elution procedure.

Time (min)	100 mM NaOH	100 mM NaOH/200 mM NaAC
0	95	5
30	80	20
30.1	60	40
45	60	40
45.1	95	5
60	95	5

### Animal Experiment

The male SD rats were obtained from the Animal Supply Center of the Zhejiang Academy of Medical Science (SCXK 2019–0002, Hangzhou, China) and fed in the SPF animal laboratory. The operations were approved by the Ethics Committee of the Zhejiang University of Technology.

Group 1 (G1) was the normal control group (NG) and Groups 2–6 were model groups (*n* = 10). Regroup G2–6 after 6 weeks of modeling induced by ACHSFD based on weight and blood pressure. In the next 7 weeks, during modeling, medicine was given at the same time. G2 was the model control group (MG, given ACHSFD); G3 received DOPS (200 mg/kg/d, p. o.); G4 received DOPS and antibiotics (DOPS + AB, p. o.); G5 received antibiotics (AB), and G6 received SCFAs (SCFAs). The protocol for ACHSFD and alcohol was adopted in our preliminary studies (Li et al., 2021). The protocol for antibiotics (metronidazole 2 g/L, azithromycin 1 g/L, and levofloxacin 0.5 g/L; p. o.) and SCFAs (butyrate 70 mM/L, propionate 30 mM/L, and acetate 20 mM/L; pH = 7.5; p. o.) were referenced to the preliminary studies partly (Zeng et al., 2020).

ACHSFD was made up of high sugar and fat diet (Nantong Trophy Feed Technology Co., Ltd., TP0800), and alcohol (4%) was given to the model groups for four consecutive days, which was increased to 8% on the sixth day and then increased by 4% every 3 days until 22%.

### Blood Pressure Measurement

Blood pressure (SBP, DBP, and MBP) was measured according to our previous studies (Li et al., 2019) by an intelligent non-invasive sphygmomanometer (Softron Beijing Biotechnology Co., Ltd., Beijing, China) every week during the research.

### Serum Biochemical Parameters

After treatment for 7 weeks, the rats’ serum was collected and serum TC, TG, HDL-c, LDL-c, ALT, and AST levels were detected by the Hitachi 7060 automatic biochemical analyzer (Hitachi, Shanghai, China) according to our previous studies (Chen et al., 2015; Lei et al., 2019).

### Detection of Serum Nitric Oxide Content

The nitrate reductase method was used to detect the NO level in the serum which is similar to our previous studies (Chen et al., 2015; Li et al., 2019).

### Histopathological Examination

Hematoxylin and eosin (H&E) and Oil Red O staining were carried out according to our previous studies (Li et al., 2019). The rats’ liver, intestine, colon, and aorta were stained with H&E. The liver tissues were stained with Oil Red O. The histopathological changes could be photographed under a biological microscope (BX43, Olympus, Japan).

### Immunohistochemistry Analysis

The expression and localization of occludin, GPCR41, and GPCR43 in the ileum and colon were determined by immunohistochemistry (IHC) as described previously (Lei et al., 2019; Li et al., 2021). Briefly, the deparaffinized tissue sections were incubated with primary antibodies or PBS overnight and washed with PBS. Then, they were incubated with horseradish peroxidase (HRP)-conjugated secondary antibody for 30 min, incubated with diaminobenzidine (DAB) chromogen, and counterstained with hematoxylin. The result could be photographed under a biological microscope (BX43, Olympus, Japan).

### Western Blotting

The expression of claudin, ZO-1, and GPCR41 in the colon was determined by Western blot as described previously (Lei et al., 2019; Li et al., 2021). In brief, the extracted proteins were separated by electrophoresis SDS-PAGE gel and transferred onto a PVDF membrane which was blocked with 5% skim milk for 2 h, followed by primary antibody incubation at 4°C overnight. Enhanced chemiluminescence (ECL) reagent was used for signal detection after secondary antibody incubation for 2 h.

### Gas Chromatography–Mass Spectrometer Analysis

The feces were collected to detect SCFAs by gas chromatography–mass spectrometer (GC-MS) analysis at the end of the experiment. Chromatography system: an Agilent DB-WAX column (30 m × 0.25 mm × 0.25 μm). Helium gas was used at the rate of 1.0 ml/min. Injection temperature, 220°C; injection volume, 1 μl; non-split injection; solvent delay time, 3.5 min. Mass spectrometry system: Electron impact ion source (EI); ion source temperature, 230°C; interface temperature, 220°C.

### 16S rRNA Amplification of V3-V4 Region and Illumina Sequencing

The fecal bacterial analysis was evaluated by 16S rRNA amplification of the V3-V4 region and Illumina sequencing as previously described (Li et al., 2019). The data were scoped on the Isanger cloud platform developed by Majorbio Bio-Pharm Technology Co., Ltd.

### Statistical Analysis

All data were one-way analysis of variance (ANOVA) expressed by mean ± standard deviation (SD). The LSD *t*-tests were applied when homogeneity of variance assumptions was satisfied; otherwise, the Dunnett *t-*test was used. A value of *p* < 0.05 was considered to be statistically significant. Diagrams were obtained by GraphPad Prism 7.0.

## Results

### Properties of *Dendrobium officinale* Polysaccharide

The total polysaccharide content of DOPS was detected by the phenol-concentrated sulfuric acid method. Glucose was taken as the standard, and the linear equation was y = 0.0404× + 0.1021 (x indicates the standard concentration and y indicates the absorbance, R^2^ = 0.9984), and the total polysaccharide content of DOPS was 54.45 ± 4.23%.

The FT-IR spectrum, which was used to assist in the structure identification of the polysaccharide (Hua et al., 2004) of DOPS (Figure 1B), showed a broad absorption band at 858.16 cm^−1^ for beta and alpha mannopyranose glycoside bond absorption peaks (Liu et al., 2017). The intense broad absorption band near 1029.99 cm^−1^ was attributed to the D-pyranose structures C-O-C and C-O-H, whereas the intense broad absorption bands near 1736.86 cm^−1^ and 3415.26 cm^−1^ were ascribed to C=O and O-H stretching vibration, respectively. Another weak peak at 2926.79 cm^−1^ was assigned to the stretching vibration of C-H of methyl.

The results of monosaccharide composition determination are described in Figure 1C. DOPS was mainly composed of mannose, glucose, and galacturonic acid with mass percentages of 61.28, 31.87, and 2.53%, respectively. The molar ratio of mannose to glucose was 1.9:1.0, indicating that mannose and glucose comprised the core active structure of DOPS, consistent with previous research (Zhang et al., 2020).

### Effect of *Dendrobium officinale* Polysaccharide on Blood Pressure in Metabolic Hypertension Rats

**FIGURE 2:**
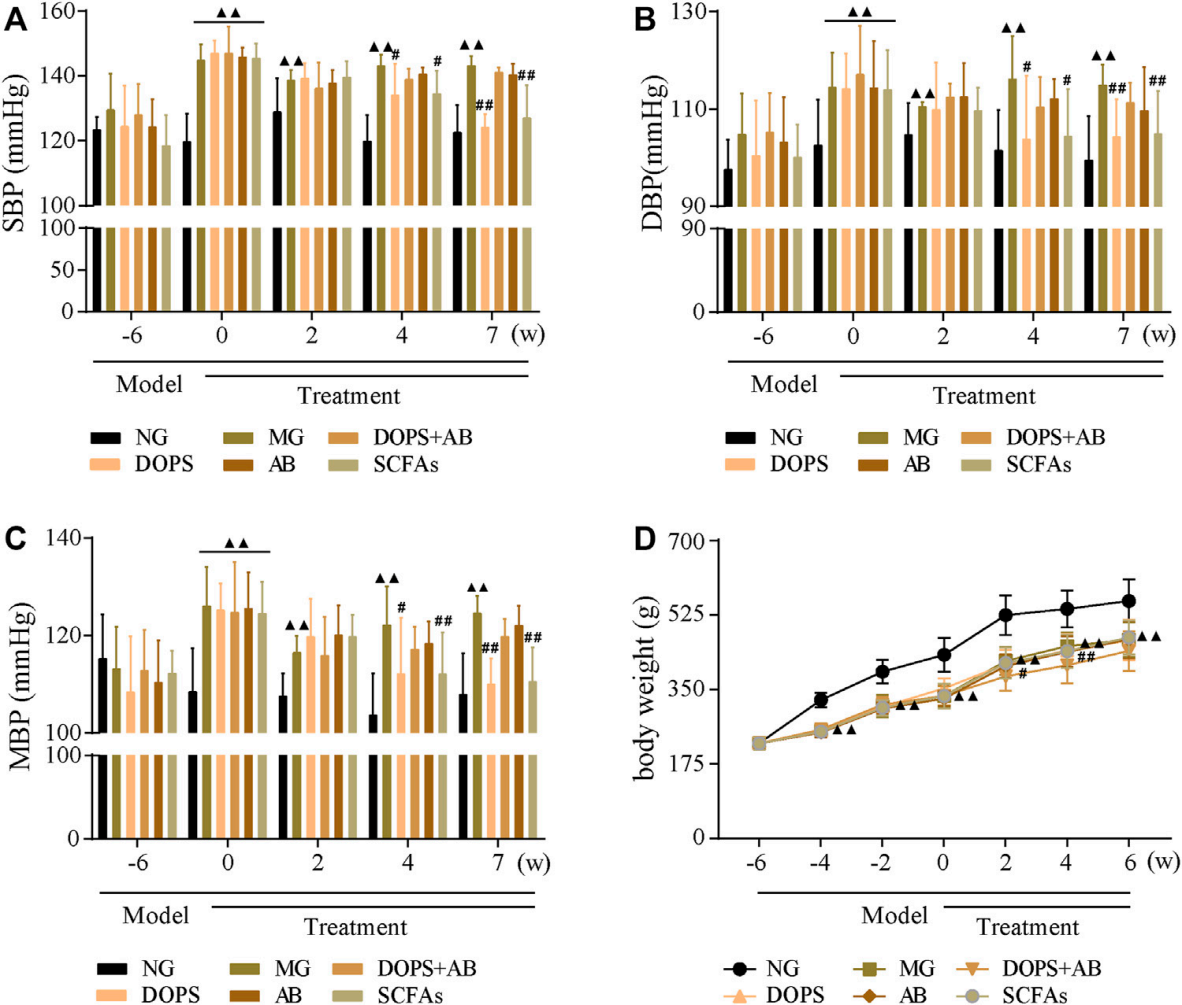
Effect of DOPS on blood pressure in the MH rats. **(A)** Changes in systolic blood pressure (SBP). **(B)** Changes in diastolic blood pressure (DBP). **(C)** Changes in mean arterial pressure (MBP). **(D)** Changes in bodyweight. NG, normal control group; MG, model control group; DOPS, *Dendrobium officinale* polysaccharide; DOPS + AB, *Dendrobium officinale* polysaccharide + antibiotics; AB, antibiotics; SCFAs, short-chain fatty acids. Compared with the normal control group, ^▲^ *p* < 0.05 and ^▲▲^ *p* < 0.01; compared with the model control group, ^#^ *p* < 0.05 and ^##^ *p* < 0.01 (*n* = 10).

Compared with the NG, the BP of MG rats was maintained at a high level after 6 weeks of modeling (*p* < 0.01) (Figure 2). After 4 weeks of administration, compared with the MG, the BP in DOPS and SCFA groups was reduced significantly (*p* < 0.01, 0.05), and the downward trend was more obvious after 7 weeks of administration (*p* < 0.01). However, throughout the entire experiment, there was no change in the BP in DOPS + AB and AB groups compared with the MG (Figure 2). Compared with the NG, the bodyweight of MH rats was reduced significantly (*p* < 0.01), which may have something to do with excessive alcohol intake. However, compared with the MG, there was no change in the bodyweight in all treated groups (Figure 2D).

The results indicated that the BP of rats fed ACHSFD was significantly increased, while DOPS had a BP-lowering effect on the model rats. The BP-lowering effect disappeared when rats were treated with antibiotics.

### Effect of *Dendrobium officinale* Polysaccharide on Serum Lipids and Liver Function in Metabolic Hypertension Rats

**FIGURE 3:**
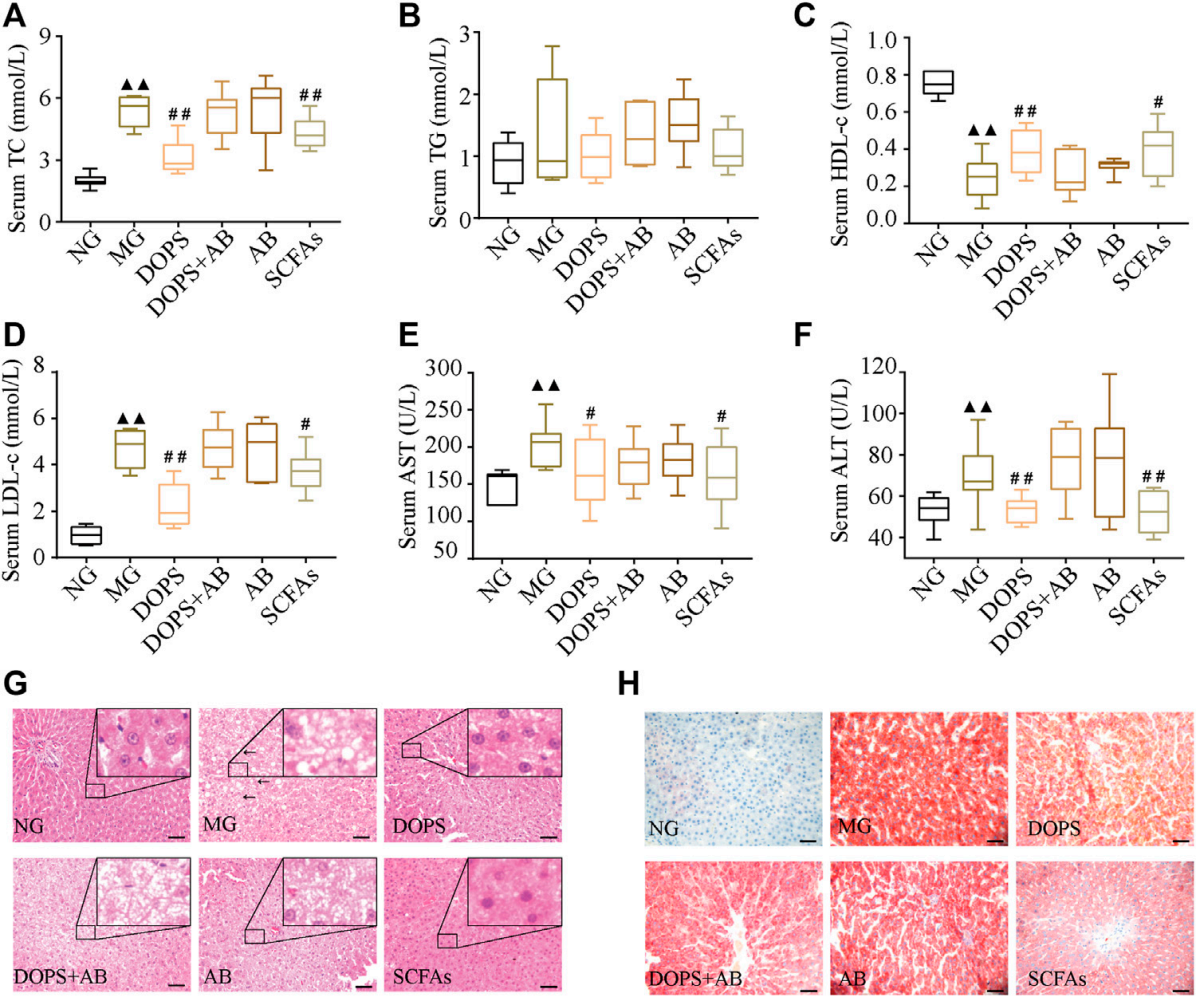
Effect of DOPS on blood lipids and liver function in the MH rats. **(A)** Changes in serum total cholesterol (TC) after administration. **(B)** Changes in serum triglyceride (TG) after administration. **(C)** Changes in serum high-density lipoprotein cholesterol (HDL-c). **(D)** Changes in serum low-density lipoprotein cholesterol (LDL-c). **(E)** Changes in serum aspartate transaminase (AST) after administration. **(F)** Changes in serum alanine transaminase (ALT) after administration. **(G)** Representative graphs of liver hematoxylin and eosin (H&E) staining (× 400). **(H)** Liver Oil Red O staining (× 400) representative graph. Bar = 50 μm. NG, normal control group; MG, model control group; DOPS, *Dendrobium officinale* polysaccharide; DOPS + AB, *Dendrobium officinale* polysaccharide + antibiotics; AB, antibiotics; SCFAs: short-chain fatty acids. The black arrows indicate liver lesions in the model control group. Compared with the normal control group, ^▲^ *p* < 0.05 and ^▲▲^ *p* < 0.01; compared with the model control group, ^#^ *p* < 0.05 and ^##^ *p* < 0.01 (*n* = 10).

After 13 weeks of modeling, compared with the NG, the serum TC, LDL-c, AST, and ALT levels were significantly increased (*p* < 0.01) (Figures 3A, D–F), and the serum HDL-c level was significantly decreased in MG rats (*p* < 0.01) (Figure 3C). Furthermore, after 7 weeks of administration, compared with the MG, the serum TC, LDL-c, AST, and ALT levels were significantly decreased (*p* < 0.01, 0.05) (Figures 3A, D–F), and the serum HDL-c level was significantly increased (*p* < 0.01, 0.05) (Figure 3C) in DOPS and SCFA groups. However, throughout the entire experiment, there were no changes in the serum biochemical indexes in DOPS + AB and AB groups compared with the MG (Figure 3).

Hematoxylin–eosin staining results showed that hepatocytes and hepatic cords were normal and arranged neatly in NG rats. After 13 weeks of modeling, compared with the NG, the liver had diffused fatty changes and inflammatory lesions in MG rats, which manifested as a few hepatocytes with vacuolar lipid droplets of varying sizes. Most hepatocytes had balloon-like changes, and the liver lobes disappeared (Figure 3G). After 7 weeks of administration, the lesions, namely, the vacuole lipid droplets, balloon-like changes, and inflammation, improved in DOPS and SCFA groups. These changes were not obvious in DOPS + AB and AB groups (Figure 3G). The Oil Red O staining results were similar to those of hematoxylin–eosin staining, especially for lipid deposition (Figure 3H). The results indicated that DOPS can inhibit MH by reversing metabolic abnormalities in model rats, and the effect could be inhibited by antibiotics.

### Effect of *Dendrobium officinale* Polysaccharide on Intestinal Pathophysiological Changes in the Metabolic Hypertension Rats

**FIGURE 4:**
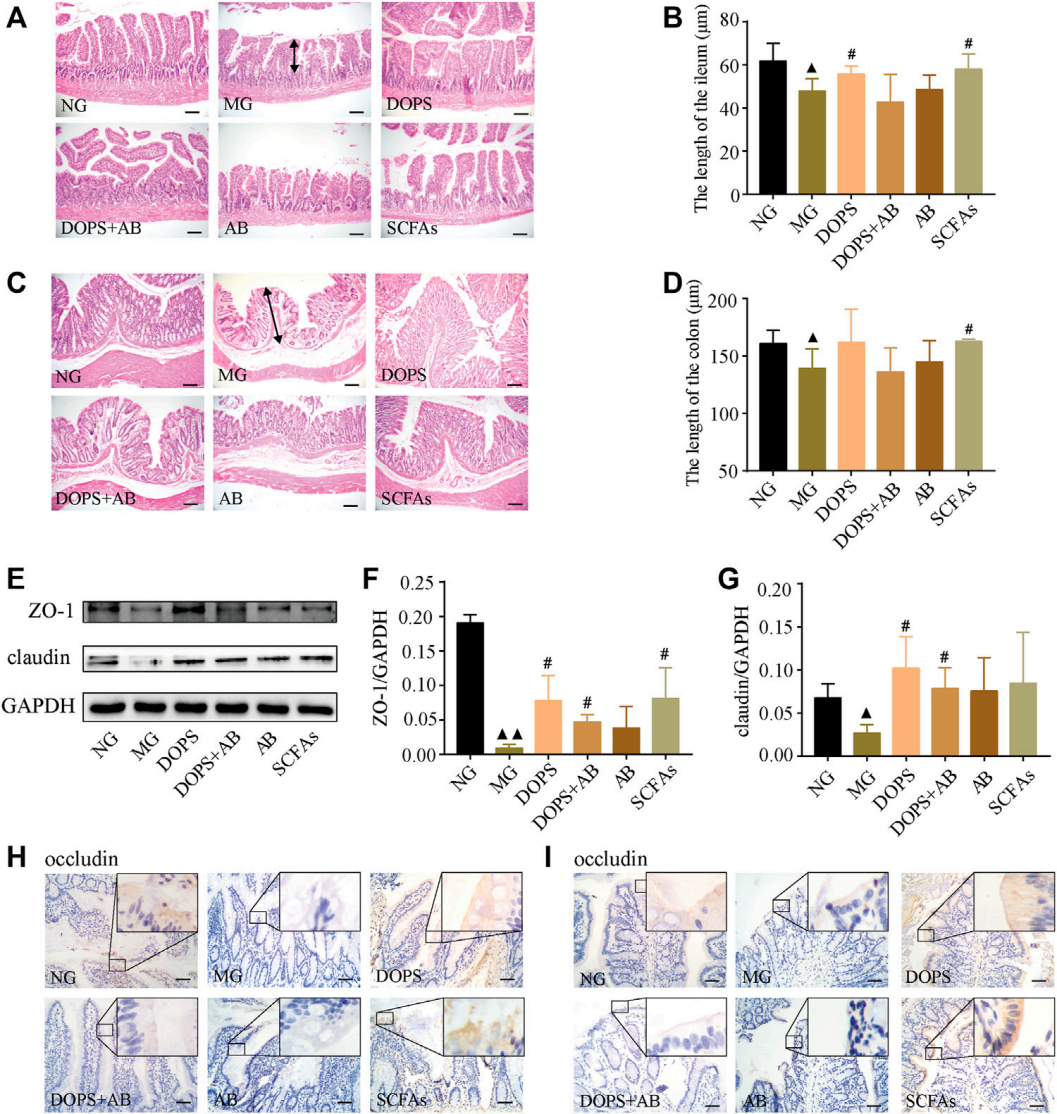
Effect of DOPS on intestinal pathophysiological changes and tight junction barrier in the MH rats. **(A–D)** Representative graphs of hematoxylin and eosin (H&E) staining and length statistics of the ileum and colon (×200), bar = 100 μm. The two-way arrows indicate the length of villi in the ileum and colon in the model group, respectively. **(E)** Representative WB graphs of ZO-1, claudin, and GAPDH in the colon. **(F,G)** Data statistics of the expression of ZO-1 and claudin in the colon (*n* = 3). **(H,I)** Representative graphs of occludin immunohistochemical staining of the ileum and colon (×400), bar = 50 μm. NG, normal control group; MG, model control group; DOPS, *Dendrobium officinale* polysaccharide; DOPS + AB, *Dendrobium officinale* polysaccharide + antibiotics; AB, antibiotics; SCFAs, short-chain fatty acids. Compared with the normal control group, ^▲^ *p* < 0.05 and ^▲▲^ *p* < 0.01; compared with the model control group, ^#^ *p* < 0.05 and ^##^ *p* < 0.01.

H&E staining was used to observe the effect of DOPS on intestinal pathophysiological changes of model rats. Compared with the NG, the ileum villi in the MG were uneven, short, loosely arranged, and partially fused, some epithelial cells and goblet cells were disordered and disappeared, and some intestinal glands were ruptured and irregularly arranged (Figure 4A). The colonic mucosa and lamina propria became shorter, part of the intraepithelial goblet cells was disorderly arranged, and glands were ruptured and abnormal (Figure 4C). Compared with the NG, the length of the ileum and colon became shorter in the MG, which significantly became longer in DOPS and SCFAs groups and had no significant changes in DOPS + AB and AB groups compared with the MG (Figures 4B, D).

WB results showed that DOPS and SCFAs could significantly increase the expression of claudin and ZO-1 in the colon compared with the MG (*p* < 0.05). Although the expression also increased in the DOPS + AB group (*p* < 0.05), the effect was also inferior to the DOPS group (Figures 4E–G). Immunohistochemical results showed that the occludin expression in the ileum and colon of the NG was more abundant. Compared with the NG, the expression was significantly reduced in the MG. Compared with the MG, DOPS and SCFAs could significantly increase the expression, while DOPS + AB and AB did not (Figures 4H, I).

The results indicated that DOPS could improve intestinal pathophysiological changes by improving the expression of intestinal tight junction proteins (TJPs) (occludin, claudin, and ZO-1) in the ACHSFD-induced MH rats.

### Effect of *Dendrobium officinale* Polysaccharide on the Fecal SCFAs and Intestinal Short-Chain Fatty Acid Transporters GPCR41/43 in the Metabolic Hypertension Rats

**FIGURE 5:**
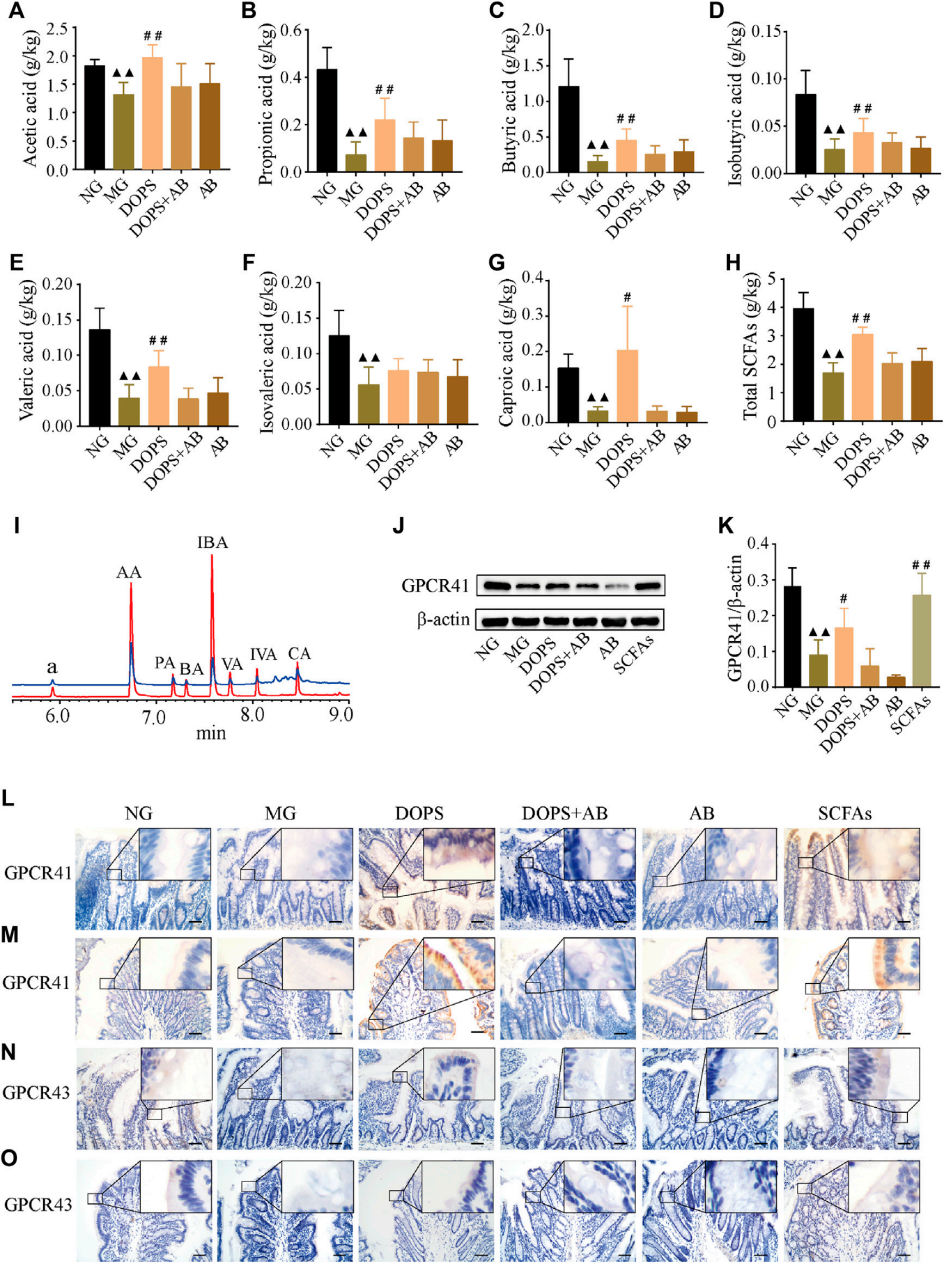
Effects on the fecal SCFAs and intestinal SCFA transporters GPCR41/43 in the MH rats. **(A–H)** Fecal acetic acid, propionic acid, butyric acid, isobutyric acid, valeric acid, isovaleric acid, and caproic acid and total short-chain fatty acid (SCFA) content (*n* = 5). **(I)** GC chromatography of sample (green line) and short chain fatty acid standard (red line); a, the internal standard of cyclohexanone, AA, acetic acid; PA, propionic acid; BA: butyric acid; IBA: isobutyric acid; VA, valeric acid; IVA, isovaleric acid; CA, caproic acid. **(J)** Representative WB graphs of the colon G protein-coupled receptor 41 (GPCR41) and β-actin. **(K)** Data statistics of the expression of GPCR41 in the colon (*n* = 3). **(L–O)** Representative graphs of GPCR41 and GPCR43 immunohistochemical staining in the ileum and colon (×400), bar = 50 μm. NG, normal control group; MG, model control group; DOPS, *Dendrobium officinale* polysaccharide; DOPS + AB, *Dendrobium officinale* polysaccharide + antibiotics; AB, antibiotics; SCFAs: short-chain fatty acids. Compared with the normal control group, ^▲^ *p* < 0.05 and ^▲▲^ *p* < 0.01; compared with the model control group, ^#^ *p* < 0.05 and ^##^ *p* < 0.01.

GC chromatography showed that SCFAs in the feces of experimental rats included acetic, propionic, butyric, isobutyric, valeric, isovaleric, and caproic acid (Figure 5I). Compared with the NG, the levels of fecal acetic, propionic, butyric, isobutyric, valeric, isovaleric, and caproic acid and total SCFA content in the MG were significantly reduced (*p* < 0.01). Compared with the MG, the levels of fecal acetic, propionic, butyric, isobutyric, valeric, and caproic acid, and total SCFA content in the DOPS were significantly increased (*p* < 0.05, 0.01) (Figures 5A–H).

WB results showed that DOPS and SCFAs could significantly increase the GPCR41 expression in the colon of the model rats and the presence of antibiotics inhibited this effect (*p* < 0.05, 0.01). Moreover, the GPCR41 expression of the DOPS group was slightly increased, which was less than that of the SCFAs group (Figures 5J, K). Immunohistochemical results showed that the expression of GPCR41/43 in the ileum and colon of the NG was abundant. Compared with the NG, the expressions were significantly reduced, which would be improved by DOPS and SCFAs. There was no such improvement effect in DOPS + AB and AB groups (Figures 5L–O). The results suggested that DOPS could improve the generation and transportation of SCFAs, while antibiotics would suppress the effect.

### Effect of *Dendrobium officinale* Polysaccharide on Aorta GPCR41-eNOS in the Metabolic Hypertension Rats

**FIGURE 6:**
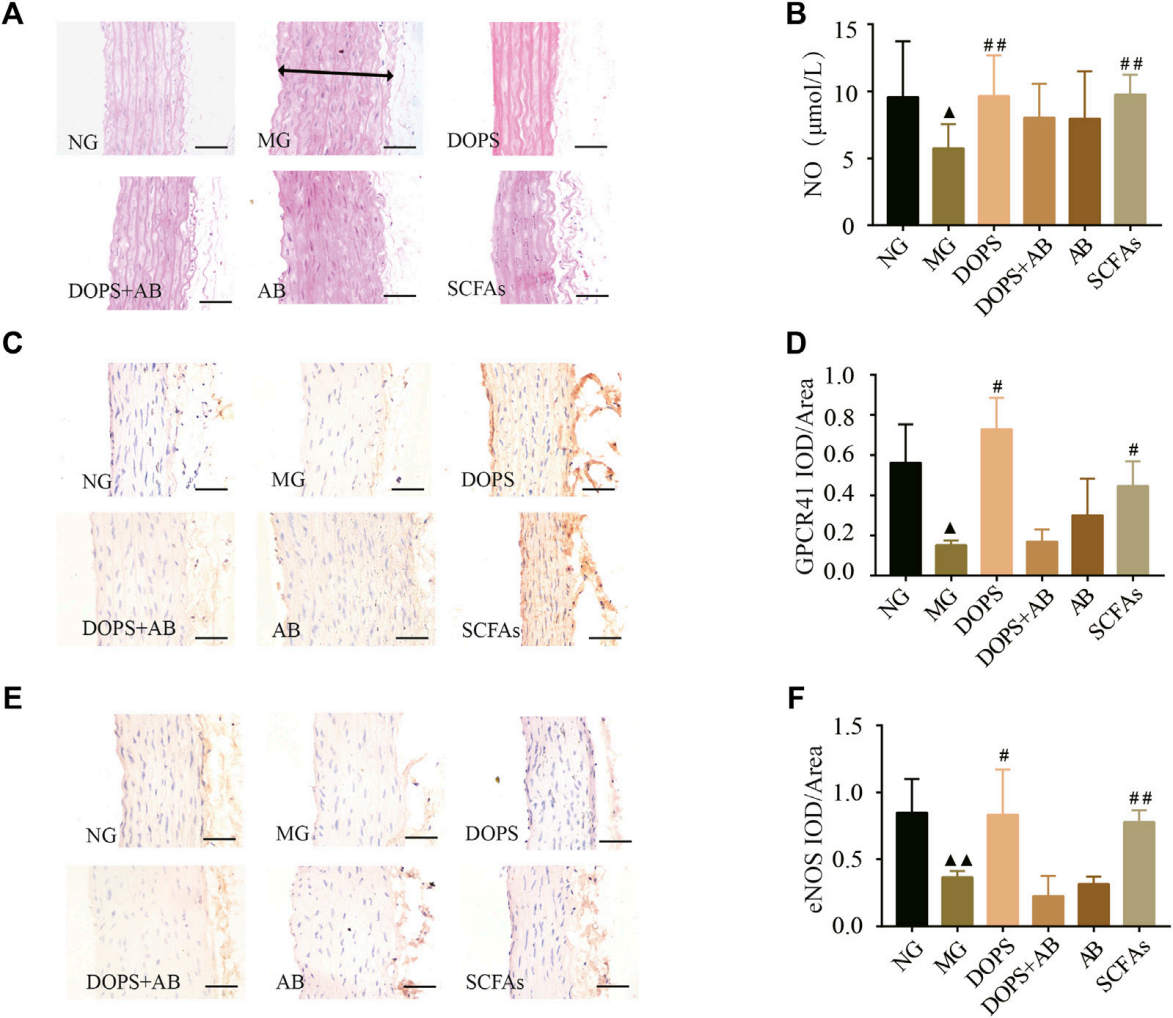
Effect of DOPS on aorta GPCR41-eNOS in the MH rats. **(A)** Representative graphs of H&E staining in the aorta (× 400). The two-way arrows indicate the thickness of the aortic arch in the model group. **(B)** The content of nitric oxide (NO) in serum. **(C)** Representative graphs of G protein-coupled receptor 41 (GPCR41) immunohistochemical staining in the aorta (×400). **(D)** Data statistics of the expression of GPCR41 in aorta. **(E)** Representative images of aorta endothelial nitric oxide synthase (eNOS) immunohistochemical staining (×400). Bar = 50 μm. **(F)** Data statistics of the expression of eNOS in aorta. NG, normal control group; MG, model control group; DOPS, *Dendrobium officinale* polysaccharide; DOPS + AB, *Dendrobium officinale* polysaccharide + antibiotics; AB, antibiotics; SCFAs, short-chain fatty acids. Compared with the normal control group, ^▲^ *p* < 0.05 and ^▲▲^ *p* < 0.01; compared with the model control group, ^#^ *p* < 0.05 and ^##^ *p* < 0.01.

In the NG, there was no intima loss or media thickening in the aorta. Compared with the NG, the aortic endothelial cells are arranged unevenly, accompanied by shedding. The media and vascular smooth muscle cells were abnormal in the MG. Compared with the MG, DOPS and SCFAs could significantly reverse these lesions in the model rats while the groups containing antibiotics could not (Figure 6A).

Immunohistochemical and NO assay results showed that the expression of GPCR41 and eNOS in the aorta and the NO content in serum were significantly reduced in the MG compared with the NG (*p* < 0.05, 0.01). DOPS and SCFAs could increase the expression of GPCR41 and eNOS and the NO content compared with the MG (*p* < 0.05, 0.01), while DOPS + AB and AB cannot (Figure 6B–F). The results showed that DOPS had an effect on aorta GPCR41-eNOS in the MH rats.

### Effect of *Dendrobium officinale* Polysaccharide on the Abundance and Phylum Level of Intestinal Flora in the Metabolic Hypertension Rats

**FIGURE 7:**
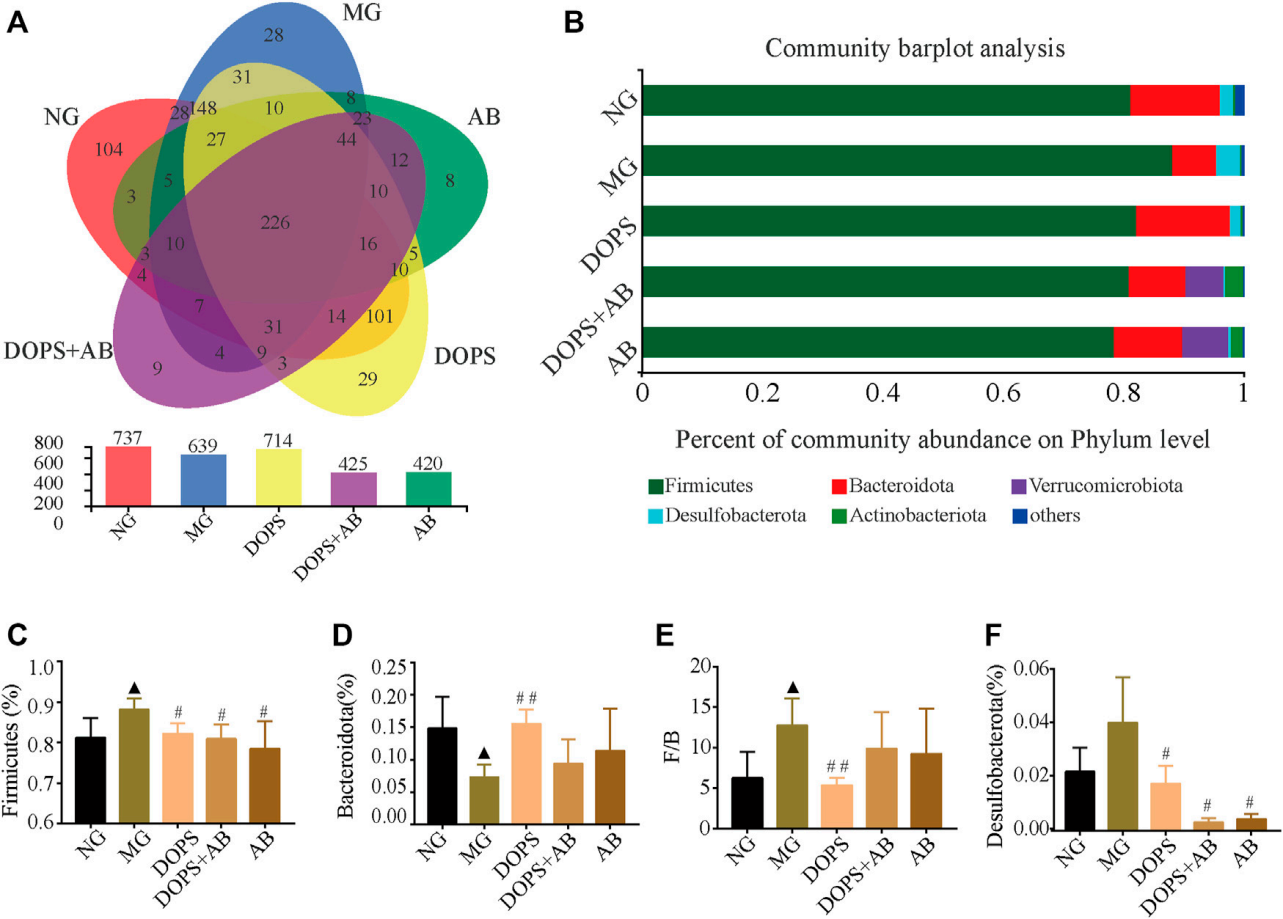
Effect of DOPS on the abundance and phylum level of intestinal flora in the MH rats. **(A)** Venn plot analysis of species on phylum level. **(B)** Percent of community abundance on phylum level. **(C–F)** Firmicutes, Bacteroidetes, and Desulfobacterota abundance ratio and Firmicutes/Bacteroidetes (F/B) statistical analysis. NG, normal control group; MG, model control group; DOPS, *Dendrobium officinale* polysaccharide; DOPS + AB, *Dendrobium officinale* polysaccharide + antibiotics; AB, antibiotics. Compared with the normal control group, ^▲^ *p* < 0.05 and ^▲▲^ *p* < 0.01; compared with the model control group, ^#^ *p* < 0.05 and ^##^ *p* < 0.01 (*n* = 3–4).

The intestinal flora was analyzed by 16S rRNA sequencing to explore the mechanism of DOPS against MH. The Venn diagram was used to analyze the operational taxonomic unit (OTU) abundance. Compared with the NG, the OTU abundance decreased in the MG; compared with the MG, the OTU abundance increased in the DOPS and decreased in the DOPS + AB and AB. (Figure 7A).

Based on the flora composition at the phylum level, the predominant phyla of rats in the NG were Firmicutes, Bacteroidota, and Desulfobacterota. Compared with the NG, the abundance of Bacteroidota in the MG was significantly reduced, while Firmicutes and the ratio of Firmicutes/Bacteroidota (F/B) were significantly increased (*p* < 0.05). Compared with the MG, DOPS could significantly increase the abundance of Bacteroidota (*p* < 0.01), decrease the abundance of Firmicutes, Desulfobacterota, and the F/B ratio (*p* < 0.05, 0.01). In addition, the abundance of Firmicutes and Desulfobacterota was decreased in the DOPS + AB and AB groups (*p* < 0.05) (Figures 7B–F). The results suggested that DOPS could significantly improve the disorder of intestinal flora.

### Effect of *Dendrobium officinale* Polysaccharide on the Genus Level of Intestinal Flora and the Correlation With SCFAs in the Metabolic Hypertension Rats

**FIGURE 8:**
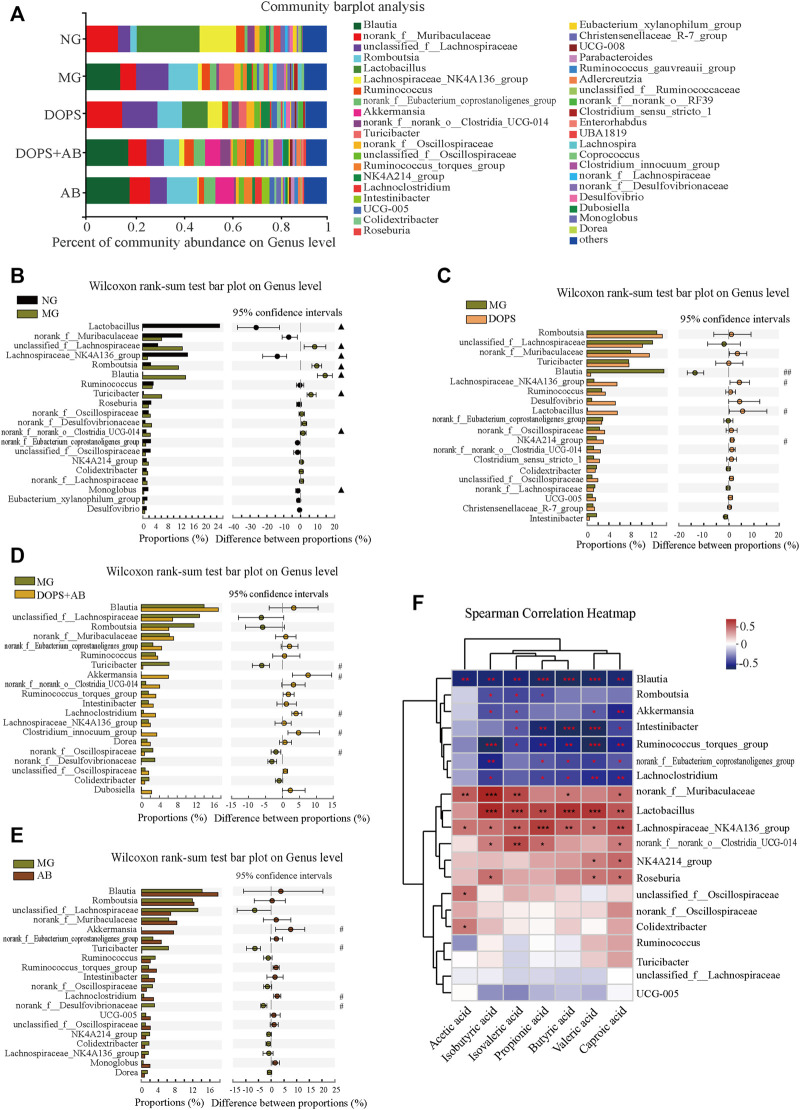
Effect of DOPS on the genus level of intestinal flora and the correlation with SCFAs in the MH rats. **(A)** Percent of community abundance on genus level. **(B–E)** Analysis of the difference of the top 20 of intestinal flora abundance between NG and MG, MG and DOPS, MG and DOPS + AB, and MG and AB on genus level. **(F)** Spearman correlation heat map analysis of intestinal flora composition and short-chain fatty acids (SCFAs) on genus level. NG, normal control group; MG, model control group; DOPS, *Dendrobium officinale* polysaccharide; DOPS + AB, *Dendrobium officinale* polysaccharide + antibiotics; AB, antibiotics. Compared with the normal control group, ^▲^ *p* < 0.05 and ^▲▲^ *p* < 0.01; compared with the model control group, ^#^ *p* < 0.05 and ^##^ *p* < 0.01; the correlation ^*^ *p* < 0.05, ^**^ *p* < 0.01, and ^***^ *p* < 0.001 (*n* = 3–4).

Furthermore, the proportion and changes of intestinal flora on the genus level were analyzed in order to clarify the relationship between intestinal flora changes and MH caused by ACHSFD and the improvement effect of DOPS. Analysis of the composition of intestinal flora at the genus level shows that the predominant genus in the NG were *Lactobacillus*, *Lachnospiraceae_NK4A136_group*, *norank_f__Muribaculaceae*, *unclassified_f__Lachnospiraceae*, etc (Figure 8A). The statistical analysis of the top 20 of the abundance of intestinal flora on genus level showed that compared with the NG, the abundance of *Lactobacillus*, *Lachnospiraceae_NK4A136_group*, and *Monoglobus* was significantly decreased in the MG, while the abundance of *unclassified_f__Lachnospiraceae*, *Romboutsia*, *Blautia*, *Turicibacter*, and *norank_f__norank_o__Clostridia_UCG-014* was increased (*p* < 0.05). (Figure 8B).

Compared with the MG, the abundance of *Blautia* was significantly decreased in the DOPS, while the abundance of *Lachnospiraceae_NK4A136_group*, *Lactobacillus*, and *NK4A214_group* was increased (*p* < 0.05, 0.01). (Figure 8C). The abundance of *Turicibacter* and *norank_f__Desulfovibrionaceae* was significantly decreased in the DOPS + AB, while the abundance of *Akkermansia*, *Lachnoclostridium,* and *Clostridium_innocuum_group* was increased (*p* < 0.05). (Figure 8D). Also, the abundance of *Turicibacter* and *norank_f__Desulfovibrionaceae* was significantly decreased in the AB, while the abundance of *Akkermansia* and *Lachnoclostridium* was increased (*p* < 0.05). (Figure 8E).

The Spearman correlation heatmap describing the relationship between intestinal flora and SCFAs showed that *Blautia*, *Romboutsia*, *Akkermansia*, *Intestinibacter*, *Ruminococcus_torques_group* etc., had significantly negative correlation with SCFAs (*p* < 0.05, 0.01, 0.001) and *norank_f__Muribaculaceae*, *Lactobacillus*, *Lachnospiraceae_NK4A136_group*, *norank_f__norank_o__Clostridia_UCG-014*, *NK4A214_group* etc., had significantly positive correlation with SCFAs (*p* < 0.05, 0.01, 0.001) (Figure 8F).

## Discussion

The primary goal of this study was to identify a bioactive component of *Dendrobium officinale* to treat MH and to explore its underlying mechanism of action. Our findings established a link between MH and DOPS. We verified that DOPS could directly modify the intestinal microbial environment and further alter the key metabolic products (SCFAs) to inhibit MH. The intestinal flora was modified by DOPS, and the abundance of Bacteroidota, which was related to the generation of acetic acid and propionic acid, was enriched (Guo et al., 2021). Meanwhile, DOPS reduced the abundance of Firmicutes and Desulfobacterota, as well as the F/B ratio, which may be related to MH (Yang et al., 2015; Zeng and Tan, 2020). Furthermore, the contents of all types of SCFAs in the feces of rats were increased, which have been demonstrated to suppress the symptoms of MH (Miyamoto et al., 2016). As such, our findings indicate that the treatment and prevention of MH by targeting the intestinal flora should take DOPS into account in the future. In addition, polysaccharides work as a whole molecule, and although many herbs and even common foods contain mannose and glucose as DOPS, their effects vary due to the proportion of monosaccharides, the way they are connected, and the size of the molecule.

In our previous study, DOFP lowered the BP in rats with MH (Li et al., 2021). In this study, DOPS, which corresponded to the total polysaccharide content of DOFP, significantly decreased the BP (SBP, DBP, and MBP) in rats with MH from the fourth week, indicating that DOPS can be used in the treatment of MH. The levels of serum lipids and liver enzymes are important indicators of health. Studies have reported that improving lipid and other metabolic abnormalities can alleviate the symptoms of MH (Li et al., 2021). Our findings show that DOPS significantly decreased the levels of serum TC, TG, and LDL-c and increased the HDL-c level in rats with ACHSFD-induced MH. In our previous study, DOFP significantly decreased the levels of serum AST and ALT. It has a certain ameliorative effect on dyslipidemia, liver function damage, liver lipid deposition, liver inflammatory lesions, and fatty changes in rats with ACHSFD-induced MH (Li et al., 2021). Furthermore, DOFP also significantly improved liver lesions in mice with non-alcoholic fatty liver disease (Lei et al., 2019). Taken collectively, these findings indicate that DOPS can improve dyslipidemia, liver function damage, and liver lipid deposition, suggesting a strong benefit for DOPS in the treatment and prevention of MH.

As important components of the intestinal mucosal barrier, tight junction proteins, including ZO-1, occludin, and claudin, are transmembrane proteins between intestinal epithelial cells (Runkle and Mu, 2013). Hypertensive rats show negative intestinal histopathological changes such as shortened intestinal villi and decreased goblet cell numbers (Santisteban et al., 2017). Furthermore, the expression of tight junction proteins decreased in hypertensive rats (Santisteban et al., 2017; Wang et al., 2021). In our previous study, pre-treatment with DOFP could reverse lipopolysaccharide-induced intestinal mucosal damage, reduce intestinal permeability, and enhance barrier function, with the expression of occludin and claudin increasing in the process (Lei et al., 2019). In this study, DOPS could improve the pathophysiological changes in the intestine by enhancing the expression of occludin, claudin, and ZO-1 in rats with ACHSFD-induced MH.

As the main metabolites generated by intestinal flora, SCFAs, such as acetate, propionate, and butyrate, are the end products of the intestinal microbial fermentation of dietary fibers and resistant starches (Koh et al., 2016). SCFAs generated in the intestine can modulate intestinal flora, as well as intestinal barrier integrity (Overby and Ferguson, 2021). They possess the bioactivity to inhibit metabolic disorders and other associated diseases such as obesity and hypertension through host receptors, including GPCRs (Hu et al., 2018). Studies have reported intestinal barrier impairment, gut dysbiosis, lower SCFA concentrations in spontaneously hypertensive rats, and the increased production of SCFAs by intestinal flora protected against hypertension-related intestinal barrier damage (Wu et al., 2019). In this study, DOPS improved the production and transportation of SCFAs, whereas antibiotics suppressed these effects. A direct effect of SCFAs on renin release and vasomotor function leading to blood pressure reduction was recently suggested in experimental hypertension, and propionate treatment shows a blood pressure-lowering effect in the hypertensive mice (Bartolomaeus et al., 2019). Clinical studies have also shown that increased circulating SCFAs concentrations were associated with reduced blood pressure and improved cardiovascular phenotypes (Chen et al., 2020). The evidence, especially for clinical evidence, in SCFAs’ clinic is relatively few. Further studies are needed to characterize the effect of SCFA supplementation to reduce blood pressure in humans.

Endothelial dysfunction is prevalent in individuals with hypertension. Endothelial NOS is suppressed in the development of hypertension, resulting in a lower endogenous NO level and vascular endothelial dysfunction, thereby leading to hypertension (Li et al., 2018). Our previous study demonstrated that DOFP could improve endothelium-dependent relaxation through the eNOS-NO pathway in ACHSFD-induced MH rats (Li et al., 2021). In this study, DOPS improved aortic lesions, increased eNOS expression in the aortic endothelium, and upregulated serum NO levels, indicating that DOPS can also activate the eNOS-NO pathway to treat and prevent MH.

As a key chemosensor in various tissues, GPCR41 is expressed in blood vessels, where it serves as a receptor for SCFAs, whose oral administration was found to alter the BP (Miyamoto et al., 2016). Studies have reported that an acute SCFA bolus decreases the BP in anesthetized mice, an effect mediated primarily *via* GPCR41 (Natarajan et al., 2016). Our previous study reported that the administration of DOFP could increase GPCR41/43 expression in the ileum and colon, as well as GPCR41 expression in the aorta of rats with ACHSFD-induced MH (Li et al., 2021). In this study, DOPS showed the same effect, indicating the importance of GPCR41/43 in the process.

Intestinal flora plays an essential role in regulating the metabolism of hosts (Hu et al., 2018). Hypertension, including MH, has been reported to associate with an imbalance of intestinal flora (Zhu et al., 2016; Li et al., 2017; Jin et al., 2019). Other studies have reported that antibiotics can disrupt the homeostasis of the intestinal flora of animals with MS and individuals with hypertension (Su et al., 2018; Zeng et al., 2020). Intestinal flora is dominated by two bacterial phyla, namely, Firmicutes or Bacteroidota, which constitute >90% of the microbes in the human gut (Zeng and Tan, 2020). The F/B ratio was significantly increased in both hypertensive animals and humans (Yang et al., 2015). Desulfobacterota is one of the main bacteria that can metabolize carbohydrates, fat, and many other compounds from food to produce H_2_S. Furthermore, the signaling and function of H_2_S in biological systems may be associated with its toxic effects (Yang et al., 2015). Studies have shown that mannose and glucose entering the intestine can change the intestinal flora, and the change of intestinal flora in this study may be caused by the uptake of mannose and glucose partially decomposed by the intestine (Zhao et al., 2014; Du et al., 2019).

In this study, DOPS could increase the abundance and diversity of the intestinal flora in model rats, especially increase the abundance of Bacteroidota and decrease the abundance of Firmicutes, Desulfobacterota, and the F/B ratio at the phylum level, which may be the main reason for the increased level of SCFAs. At the genus level, DOPS significantly increased the abundance of beneficial genera such as *Lactobacillus* and *Lachnospiraceae_NK4A136_group*, whereas antibiotics significantly increased the abundance of harmful genera such as *Lachnoclostridium* and decreased that of beneficial genera such as *Turicibacter* and *norank_f_Desulfovibrionaceae*. The Spearman correlation heatmap revealed that SCFAs were correlated with the genera such as *Blautia*, *Romboutsia*, *Akkermansia*, *Intestinibacter*, and *Ruminococcus_torques_group*.

**FIGURE 9:**
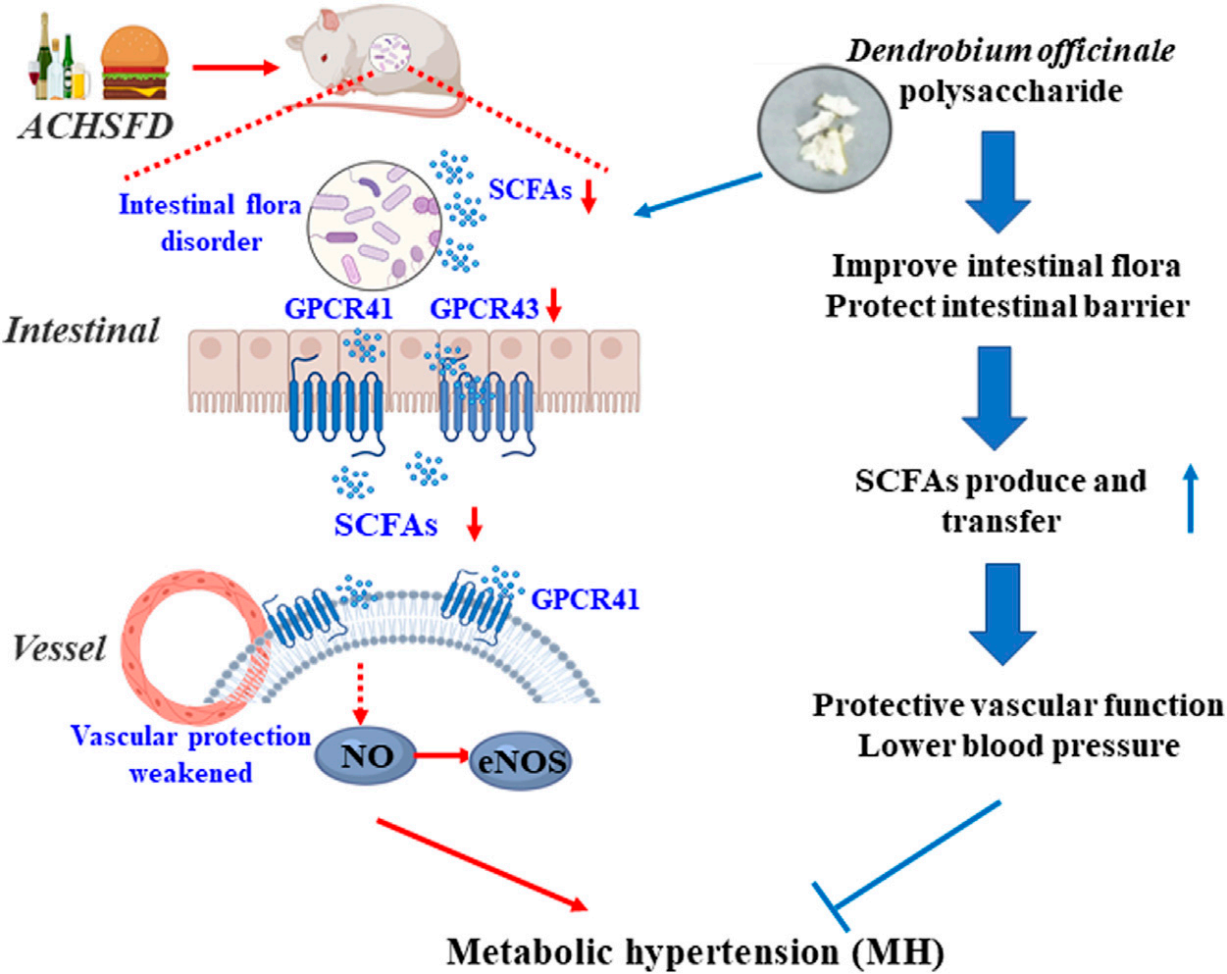
Graphical abstract: DOPS treatment with the metabolic hypertension rats by activating the enteric-origin SCFAs-GPCR43/41 pathway.

In summary, DOPS is the active component of *Dendrobium officinale*, which improved the intestinal flora and increased the intestinal SCFAs, thereby improving vascular function and lowering the BP in the rat model of MH. However, these effects were all reversed by antibiotics. These findings indicate that DOPS, the active component of *Dendrobium officinale*, has beneficial effects on rats with MH by activating the intestinal SCFA-GPCR43/41 pathway (Figure 9). This study also had limitations. Polysaccharides should be further purified and structurally identified to determine their efficacy. Also, the effects of the related intestinal flora and SCFAs should be further validated.

## Data Availability

The datasets presented in this study can be found in online repositories. The names of the repository/repositories and accession number(s) can be found below: https://www.ncbi.nlm.nih.gov/bioproject/846193.

## Abbreviations

AB, antibiotics; ACHSFD, excessive alcohol intake and high-sugar/high-fat diet; ALT, alanine transaminase; ANOVA, one-way analysis of variance; AST, aspartate transaminase; BP, blood pressure; DO, *Dendrobium officinale*; DOFP, *Dendrobium officinale* ultrafine powder; DOPS, *Dendrobium officinale* polysaccharide; DOPS + AB, *Dendrobium officinale* polysaccharide + antibiotics; eNOS, endothelial nitric oxide synthase; FT-IR, Fourier transform infrared spectroscopy; GC-MS, gas chromatography–mass spectrometer; GI, gastrointestinal; GPCR, G protein-coupled receptor; H&E, hematoxylin and eosin; HDL-c, high-density lipoprotein cholesterol; HPLC, high-performance liquid chromatography; LDL-c, low-density lipoprotein cholesterol; MH, metabolic hypertension; MG, model control group; NG, normal control group; NO, nitric oxide; OUT, operational taxonomic unit; SCFAs, short-chain fatty acids; SPF, specific-pathogen-free; TC, total cholesterol; TCM, traditional Chinese medicine; TG, triglyceride; ZO-1, zonula occludens protein 1.
